# Wastewater pandemic preparedness: Toward an end-to-end pathogen monitoring program

**DOI:** 10.3389/fpubh.2023.1137881

**Published:** 2023-03-21

**Authors:** Justin R. Clark, Austen Terwilliger, Vasanthi Avadhanula, Michael Tisza, Juwan Cormier, Sara Javornik-Cregeen, Matthew Clayton Ross, Kristi Louise Hoffman, Catherine Troisi, Blake Hanson, Joseph Petrosino, John Balliew, Pedro A. Piedra, Janelle Rios, Jennifer Deegan, Cici Bauer, Fuqing Wu, Kristina D. Mena, Eric Boerwinkle, Anthony W. Maresso

**Affiliations:** ^1^TAILOR Labs, Baylor College of Medicine, Houston, TX, United States; ^2^Department of Molecular Virology and Microbiology, Baylor College of Medicine, Houston, TX, United States; ^3^Alkek Center for Metagenomics and Microbiome Research, CMMR, Baylor College of Medicine, Houston, TX, United States; ^4^UTHealth Houston School of Public Health, Houston, TX, United States; ^5^Texas Epidemic Public Health Institute (TEPHI), UTHealth Houston, Houston, TX, United States; ^6^Center for Infectious Diseases, Department of Epidemiology, Human Genetics and Environmental Sciences, Houston, TX, United States; ^7^El Paso Water Utility, El Paso, TX, United States; ^8^Pediatrics Department, Baylor College of Medicine, Houston, TX, United States; ^9^The University of Texas Health Science Center at Houston, Houston, TX, United States; ^10^Department of Biostatistics and Data Science, UTHealth School of Public Health, Houston, TX, United States; ^11^Human Genetics Center, Department of Epidemiology, Human Genetics and Environmental Sciences, Houston, TX, United States

**Keywords:** wastewater, virus, pathogens, detection, epidemiologic, public health, early warning system

## Abstract

Molecular analysis of public wastewater has great potential as a harbinger for community health and health threats. Long-used to monitor the presence of enteric viruses, in particular polio, recent successes of wastewater as a reliable lead indicator for trends in SARS-CoV-2 levels and hospital admissions has generated optimism and emerging evidence that similar science can be applied to other pathogens of pandemic potential (PPPs), especially respiratory viruses and their variants of concern (VOC). However, there are substantial challenges associated with implementation of this ideal, namely that multiple and distinct fields of inquiry must be bridged and coordinated. These include engineering, molecular sciences, temporal-geospatial analytics, epidemiology and medical, and governmental and public health messaging, all of which present their own caveats. Here, we outline a framework for an integrated, state-wide, end-to-end human pathogen monitoring program using wastewater to track viral PPPs.

## Historical backdrop

“A sewer is a cynic. It tells everything.” Victor Hugo, *Les Miserables* (1892).

In 1939, a plane departed Detroit in route to Connecticut carrying a very unusual piece of cargo. Destined for the laboratories of Drs. John Paul and James Trask at Yale University, the package consisted of several samples of Detroit city sewage. Years earlier, Paul and Trask reasoned that since the virus that causes polio could be found in fecal matter [first reported in 1912 (1)], it may also be shed into city wastewater (2). When macaques were injected with the sewage (there was no PCR at the time and microscopy was still developing) showed signs of poliomyelitis, a result confirmed by researchers in Stockholm (3), it suggested a human virus found in public excrement might report on the state of disease at the population level. However, it was a mentee of Paul, Dr. Joseph Melnick, who showed in the 1940s that polio levels in stool and sewage are associated with the number of severe cases in the population (a result that prompted him to search for viral prevalence based on fecal shedding rates), studies that birthed the field of wastewater-based epidemiology (WBE) (1, 4, 5). This led to the implementation of environmental poliovirus surveillance (EPS) systems in countries where polio cases are still endemic, and the emergence of WBE for poliovirus and other pathogens (6).

## Recent SARS-CoV-2 experience—Rebirth

Success of the use of wastewater to monitor SARS-CoV-2 levels, and forecasting future trends aiding in public preparation and hospital-readiness, has reinvigorated viral WBE. Consensus is that similar approaches may be applied to other human viral pathogens, including adenoviruses, enteroviruses, noroviruses, rotavirus, and hepatitis viruses. The Centers for Disease Control and Prevention (CDC) initiated the National Wastewater Surveillance System (NWSS) in September 2020 to track the dispersion of SARS-CoV-2. Our own team's activity began in April of 2020 in the cities of Houston and El Paso, Texas, both of which have implemented a city-wide SARS-CoV-2 wastewater (WW) monitoring program (7–9). Recently, the CDC expanded their SARS-CoV-2 wastewater testing program to include poliovirus after vaccine derived poliovirus was detected in New York state (10). This implies a U.S. readiness to apply such a program and science to other viral pathogens.

SARS-CoV-2 and other respiratory viruses are well-known to cause gastrointestinal (GI) symptoms like diarrhea and vomiting (11–16). The GI manifestations are associated with viral RNA and infectious virus in fecal samples and can be detected throughout the course of infection (17–19). Additionally, histologic studies have shown SARS-CoV-2 virions damaging the GI epithelium (17, 19, 20). SARS-CoV-2 infected persons have peak viral loads 1–3 days before symptom onset and can shed virus for three or more weeks (21, 22). Both symptomatic and asymptomatic individuals can transmit the virus efficiently and can have prolonged viral shedding (23–25). Although it cannot be expected that all human viruses will present with an infection biology and natural history that is conducive to WBE (such as SARS-CoV-2), there is reason to believe many viruses like adenoviruses and enteroviruses with major epidemic potential will be amenable to similar WBE. This is the impetus for the efforts described in this perspective.

To our knowledge, comprehensive province/state or nationwide monitoring of various human pathogens in wastewater has not been implemented anywhere. Even the monitoring of illicit drug use, which was proposed by the U.S. Environmental Protection Agency 2 decades ago (though it is more commonly used in European cities), appears to have no standardized widespread use (26, 27). On the city level, SARS-CoV-2 has been monitored in wastewater in every continent except Antarctica (8, 28–32). The closest any country has come to implementing SARS-CoV-2 wastewater monitoring on the national level may be, as described above, the National Wastewater Surveillance System (NWSS) in the U.S. by the Centers for Disease Control and Prevention and the U.S. Department for Health and Human Services in September 2020 (33). Most of the recently published WBE studies appear to be the work of academic researchers analyzing samples they have been given access to or local health departments (or equivalent) working with academic researchers to monitor SARS-CoV-2. The NWSS was started to coordinate these SARS-CoV-2 wastewater monitoring efforts in the U.S. What started with pilot sites in 8 states in 2020 now has over 1,250 sites covering over 100 million people (33). However, the NWSS Committee on Community Wastewater-Based Infectious Disease Surveillance points out a major shortfall with the NWSS in that it currently “…consists of localities, tribes, and states that were willing and able to participate during the pandemic emergency…” and this pandemic emergency, “…spurred many researchers and utilities to volunteer their labor and donate resources in support of the effort, but the vision of a sustained national wastewater surveillance system necessitates a shift from volunteerism to a strategic national plan with well-defined roles supported by federal investments” (33). Given the success of the NWSS in tracking SARS-CoV-2 and citing success in tracking vaccine-derived polio outbreaks in London and New York, and success in rapidly tracking monkeypox, the aforementioned committee has recommended expanding the NWSS efforts to monitor other human pathogens (10, 33–37).

Reminiscent of the NWSS in the U.S., national and international efforts to track SARS-CoV-2 have recently been made in Israel and the European Union, respectively. In Israel, researchers at the Israel Ministry of Health have described their methods of tracking SARS-CoV-2 and SARS-CoV-2 variants in wastewater using PCR on samples from 13 treatment plants that cover more than 50% of Israel's population (38). In 2021, the European Commission adopted a Recommendation that EU Member States work toward monitoring SARS-CoV-2 in wastewater (39). As of March 2022, more than 1,370 wastewater treatment plants are under surveillance (40). However, to our knowledge, no effort to expand these efforts to other pathogens in a routine manner has been made.

## A statewide pandemic preparedness initiative

In the Spring of 2021, the 87^th^ Texas Legislature established the Texas Epidemic Public Health Institute (TEPHI). Housed within The University of Texas Health Science Center at Houston (UTHealth Houston), TEPHI's mandate is to work collaboratively with state, local, and federal agencies, academic institutions, professional associations, businesses, and community organizations to better prepare the state for public health threats. TEPHI's mission and structure is informed by lessons learned during the Texas response to the COVID-19 pandemic to address gaps in public health organization and infrastructure in order to better inform, train, and protect Texans.

**Figure 1:**
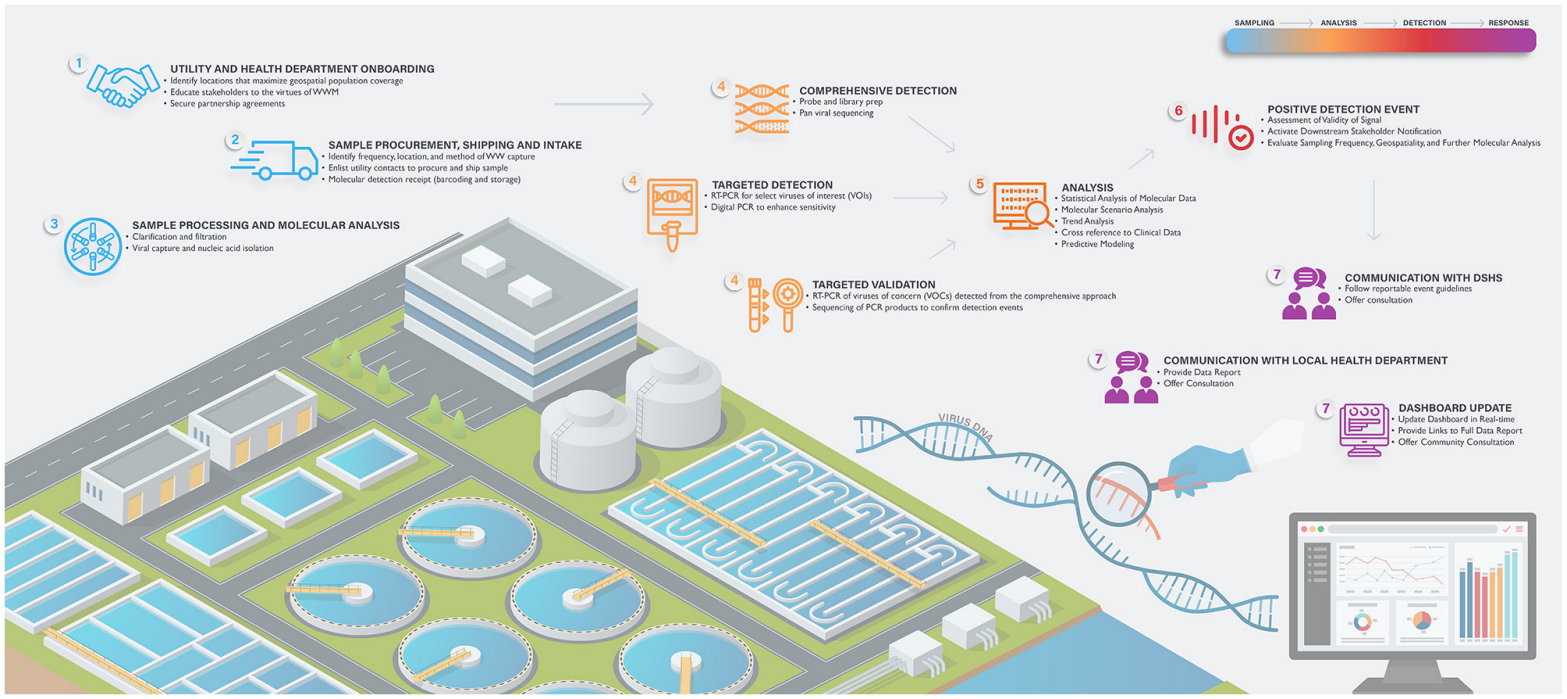
Formation of an end-to-end, statewide wastewater viral epidemiology program. Step 1—utility onboarding; Step 2—sample procurement, shipping and intake; Step 3—sample processing and molecular analysis; Step 4—targeted and comprehensive detection with targeted validation; Step 5—analysis; Step 6—positive detection response; Step 7—health department notification, communication to government stakeholders and community data update.

As part of the effort, TEPHI is launching numerous programs to support community preparedness across the state, including a collaboration with Baylor College of Medicine (BCM) and the UTHealth Houston School of Public Health (SPH) to establish a statewide Texas Wastewater Environmental Biomonitoring (TexWEB) network. The TWC (TEPHI Wastewater Consortium) will (1) partner with state utilities and public health departments to promote the virtues of wastewater science; (2) establish standard operating procedures (SOPs) for the detection of viral and/or other pathogen nucleic acid from complex wastewater sludge; (3) incorporate the latest cutting-edge technologies to enhance target detection; (4) serve as the state's real-time and ongoing wastewater pathogen monitoring system (prioritizing disease-causing human viruses in the first stages); (5) generate health department, health care and community data repositories that allow users to assess risks and trends in their counties; and (6) establish an effective chain-of-command reporting network that informs public health departments and state governments concerning levels and trends of viral PPPs in sentinel communities across the state. Charges 5 and 6 are particularly important, specifically for ensuring agreement when a detection event poses an immediate or sustained health concern, and the processes by which stakeholders are notified. This article provides an overview of the planning and effort for the first 6 months of the program, hopefully serving as a guide for other states to consider implementation of similar monitoring endeavors. Figure 1 demonstrates the specific procedural and methodological elements of this program, which are outlined in detail below.

## Nucleic acid: The universal viral WBE detection target

The hallmark principle of WBE is to translate the upstream detection of a chemical or biological substance into reasonable public health information or action. Some of the earliest WBE involved monitoring for pollutants in the early 20^th^ century in England (41, 42). Contemporary WBE has expanded to include pesticides (43) macrolide antibiotics (44), organophosphate esters (45), illicit substances (46–55), antibiotic resistance (56–58), and the topic herein, pathogenic viruses. Regardless of what is detected, all detection activities are unified around the assumption that the concentration of a substance, biologic (here a virus) and/or other agent will be proportional to the amount excreted by the population or contaminating the water supplies. The levels of this agent are thought then to be reflective of the relative risk or status of the health of the population that sheds or is exposed to the agent.

To this point, most non-pathogen WBE has been based on chemical analysis of small molecules or chemical substances. Since each substance has its own unique chemical characteristics, the method by which the agent is detected must be tailored to utilize these properties. This dilemma is potentially less of a concern for viral WBE for two main reasons, which, in theory, may facilitate the monitoring of many viruses of concern using a shared or single methodological platform. These features are (1) the universal presence of nucleic acid (DNA and RNA) in all pathogens, including viruses and (2) the invention of oligo-based priming and amplification of said DNA by polymerase chain reaction (PCR) or of RNA by the reverse transcription of RNA into DNA with subsequent amplification of the DNA target (RT-PCR). The first feature simplifies detection technology to a narrow chemical space (nucleic acid, being composed of only four bases, is much more chemically similar to all other nucleic acid, regardless of the source pathogen). The second feature facilitates the massive amplification of trace amounts of this molecule to greater than a trillion times its original concentration, thereby enhancing sensitivity. The high specificity of primers matched to the target in question and the measurement of light-emitting probes bound to amplified products (RT-PCR) generates a highly sensitive DNA/RNA detection system, regardless of pathogen. When also applied to modern DNA sequencing technologies to derive a genetic barcode of the amplified nucleic acid, the unambiguity of sequence information makes identification of the pathogen unequivocal. As such, these principles and technologies are/have been used detect the presence of viral and bacterial pathogens, fecal bacteria, and antibiotic resistance genes (59–65).

## The Consortium's methodological approach to viral detection

In the WBE efforts proposed herein, the Consortium has leveraged the above principles to implement two methodological approaches to track human viruses in wastewater (Supplementary Figure 1). The first (so-called “targeted” method) uses RT-PCR and Digital PCR to detect respiratory, gastrointestinal, and blood-borne viruses that are either commonly transmitted in community settings (e.g., SARS-CoV-2, Flu, RSV, norovirus) or are periodically endemic to the Southern part of the U.S. (some arboviruses, including dengue viruses). The advantage of a targeted approach is sensitivity and speed. By using well-designed primers to the virus in question and a validated PCR assay, very low levels of viral nucleic acid can be detected in hours. Digital PCR increases sensitivity by diluting the sample into hundreds to thousands of partitions, thereby ensuring that inhibitors of a reaction may be “diluted out” of some partitions. The technique is useful for detecting nucleic acid that may be in low abundance for one reason or another (see below).

PCR contrasts with the second method, termed by the Consortium “agnostic” or “comprehensive.” Past efforts to characterize the virus metagenome (all the genomes of viruses in a sample) have relied on enrichment of virus-like particles, the capture of viral nucleic acid with probe-based pulldowns, or shotgun whole genome sequencing (66–69). The preponderance of plant viruses or phage in these datasets, and/or the use of probes designed for only a subset of human viruses, limits a pan assessment of disease-causing human viruses. The agnostic approach used by this Consortium employs a next-generation probe-based capture sequencing panel (TWIST Comprehensive Viral Research Panel) capable of detecting over 3,000 human and animal viruses, as well as novel variants of known viruses targeted by the panel. Additionally, while the enrichment step utilized prior to next-generation sequencing vastly lowers the amount of off-target sequencing generated from other WW components, the sheer number of detectable viruses necessitates deep sequencing to achieve both the breadth and depth of reads needed for reliable viral detection. While the cost of sequencing continues to decrease year-over-year, the agnostic approach is more expensive than the targeted approach. Importantly, whereas the agnostic approach might have reduced sensitivity as compared to the targeted approach, its value is that it provides an unbiased “whole virome” analysis of a complex sample. This allows the Consortium to capture “everything else” not covered by the targeted analysis, which may prove useful for hundreds of other viruses that cause human diseases, variants of specific viruses (because sequence information increases specificity), as well as novel emerging viruses that are not yet on any clinical radar. Unlike the targeted approach, the agnostic method takes a few weeks for a full analysis to be finished, incurs additional costs, and requires significant technical expertise. A summary of advantages and disadvantages to each technique outlined here can be found in Supplemental Table 1.

## Validation and limitations of current methodological approaches

Although nucleic acid provides for streamlining of the detection pipeline, there are recurring concerns that one must be aware of and require further investigation. We briefly discuss some here so that others who wish to consider this work are informed. Because each virus has its own biology, chemistry, and natural history of infection, each of these attributes will affect viral levels detected in WW. Some overriding determinates are that the virus or its nucleic acid (1) must be shed in human excrement (or enter wastewater in some consistent way); (2) must be relatively stable in raw sewage exposed to a harsh chemically and environmentally-shifting conditions; and (3) be enriched during the viral capture steps. Many viruses have a human infection biology that likely precludes them from excretion into the WW (perhaps their infection tropism has nothing to do with the gastrointestinal or urinary tract). Even if excreted, others have a capsid or membrane structure that is unstable, thereby exposing their sensitive nucleic acid to harmful sludge conditions. Finally, viruses may be excreted and be stable but if the targeted or capture method fails to bind them, they cannot be detected. Thus, any pipeline attempting to provide universal (or even highly targeted) detection may have one or more of these issues affecting the outcome.

When conditions 1–3 above are met, other factors may limit sensitivity or reproducibility. Adding to the list above, these include, but are not limited to; (4) the number of infected people; (5) the amount or frequency of shedding; (6) transit time to the plant; (7) composition of the plant sewage (8) environmental changes such as rainfall or temperature; (9) collection technique and sample transport; (10) storage of sample; (11) capture technique (e.g., size, ionicity); (12) co-purifying inhibitors of the capture or detection methods (including non-specific binding of viral material to wastewater matter or direct inhibition of this matter of downstream processes); (13) whether the liquid or solid phase is examined; and finally (14) sensitivity, specificity, and genome coverage of the probes and primers, homology of the primers to variants and emerging pathogens, and so forth. A summary of limitations and associated reasons that impact viral and pathogen detections can be found in Supplemental Table 2. Where possible, care should be taken to limit the negative impact of these on the process to limit attrition of signal. In the targeted approach, this is easier to achieve because the emphasis is on a single virus. Use of the targeted virus or its nucleic acid as a proxy (“spike-in”) during tests of the pipeline can determine what factors affect levels and signal (attrition). However, in the agnostic approach, because of the sheer magnitude of total viruses surveyed, it is not currently possible to optimize capture, amplification, and detection for every virus. In these cases, a best-fit approach is taken, whereby what works for a subset of key viruses (we use SARS-CoV-2 as a representative constituent) provides confidence that the methodological conditions are conducive to detecting many but not all viruses.

How will detection events be validated? The PCR technology itself has shortcomings in that nucleic acid shearing can reduce priming (false negative), while the lack of primer specificity can produce off-target amplification (false positive). One common method for validating the targeted method (here PCR) is to have the test repeated in a second, independent laboratory (70, 71). However, a second laboratory testing the same corrupted sample may produce the same result. One benefit of using a two-pronged approach (i.e., targeted and agnostic as described above) is the orthogonal nature of verification; if two distinct tests are positive, and detection is achieved *via* different methods, there is strong evidence the signal is real. In time, we hope to add a third assay, so-called “targeted sequencing,” which will employ primers that tile across the entire viral genome of the virus in question, to produce sequence information that coverages at or near 100% of the genome. Not only does this provide confidence the detection is real, it also has the value of increasing the ability to identify variants that are present or emerging (72–74).

## Onboarding utilities and safety

Successful upstream of viral detection requires public works utilities to gain access to wastewater samples and expertise in wastewater treatment processes and engineering. Specific legal agreements regarding disclosures of sampling sites, use of the information, and general risk assessment may be required between those analyzing the samples and those providing them. There are costs associated with sampling, including personnel, equipment needed for sampling, and shipment of the sample itself. Many utilities already understand the risks associated with working with such material, but detection science may reveal additional pathogens not routinely considered for risk assessment. Quantitative microbial risk assessment (QMRA) is a systematic approach to estimating the probability or likelihood of infection, illness and death from exposure to disease-causing pathogens. The dynamic, four-component framework of hazard identification, dose-response assessment, exposure assessment, and risk characterization defines an iterative process that comprehensively evaluates the pathogen-host interaction (75). The focus of a QMRA is the pathogen, with data generated from field and/or laboratory studies to inform its occurrence in the environment, its survivability and virulence properties, and its transmission pathways. Previous human dose-response studies are available for many pathogens transmitted through environmental sources (such as air, water, food and fomites), and best-fit mathematical models have been developed to represent infection probabilities for specific microorganisms (76).

One of the objectives of our TWC pipeline is to develop a reverse QMRA to public health readiness, for example to estimate the number of infections in a community based on viral levels in WW. The data obtained in a traditional QMRA is pathogen specific with information characterizing the host-pathogen interaction including incubation period, morbidity ratios, range of symptoms, likelihood of secondary transmission, and specific sequelae, such as excretion patterns. By applying the appropriate dose-response model in a reverse QMRA, the number of infections within a community can be estimated based on the microbial composition of the sewage serving that municipality. This QMRA approach can be used to interpret wastewater monitoring trends observed over time, with qualitative characterization revealing unseasonal pathogens due to unexpected community infections and illnesses. Algorithms can then be developed to estimate the number of community infections after a PCR or sequencing result is attained. Through integration, the qualitative and quantitative QMRA output can estimate the likelihood of community transmission, determine whether an outbreak is occurring, or estimate if an epidemic is imminent. At the moment, reverse QMRA seems possible for SARS-CoV-2, influenzae, norovirus, monkeypox, and possibly respiratory syncytial virus (RSV). Such output will address the assumption described earlier that the amount of pathogen detected in sewage is proportional to the amount excreted by the population served by that wastewater system, as well as help estimate the possible number of people infected.

## Data analysis and statistics

It has been reported that SARS-CoV-2 detection in wastewater leads case reports by 2–14 days, though it has been argued that a 4-day lead time is the most plausible (8, 77, 78). Estimations of lead times for hospitalizations have been reported to be 4–8 days (79, 80). Though limitations of such WBE lead time calculations have been noted, it has been calculated for other pathogens such as influenza virus A, where wastewater detection led clinical case detection by 17 days, and RSV, where wastewater detection led clinical detection by about 1 week (77, 78, 81). Olesen et al. (77) succinctly argued some of the issues with the currently ill-defined idea of “lead times” in WBE and how the term has been used in different circumstances. They outlined these circumstances as, (1) “qualitative detection of disease presence/absence,” (2) “independent, quantitative estimate of community-level disease,” and (3) “quantitative estimate of rapid changes in disease incidence” (77). For the purposes of TEPHI, the first circumstance—“qualitative detection of disease presence/absence”—is the initial goal when dealing with non-SARS-CoV-2 pathogens.

Essential to viral WBE is the formation of predictive or forecasting models that provide lead-time warning of outbreaks, transmission, or an ongoing epidemic or pandemic. Formation of such models requires data analysis on historical data with both wastewater and epidemiological/clinical data to establish the relationships between viral nucleic acid detected in the wastewater and the epidemic severity (e.g., reported case rates), from which models may be further developed for forecasting. As an example, various studies have reported the promising potentials of WBE during the SARS-CoV-2 pandemics, however challenges remain. A recent review by Faraway 2022 made recommendations in building quantitative prediction models using WBE (82), emphasizing key factors that determine the accuracy of any forecasting models including: (1) sampling design; (2) sensitivity and reproducibility of the measurement process; (3) availability of the auxiliary environmental variables; (4) the amount of clinical data to provide for cross correlation; and (5) prior knowledge of the particular disease under study (more difficult for newly emerging pathogens, for example Zika). All of these are being addressed by the TEPHI Wastewater Consortium in some fashion. In addition, we identified other obstacles as: (6) the lack of state and/or countrywide data repositories accessible to interested stakeholders; (7) fragmented clinical data sets that lack metadata; and (8) lack of robust real-time data sharing portals (not only among investigators in the same fields but also in distinct fields, example linking viral PCR data to nasal testing data for SARS-CoV-2).

## The Consortium's public health approach to viral WBE

Equally challenging as viral detection is the use of this information for public health action. Although the practice is still developing, some broadly agreed-upon actions are emerging. At this juncture it is important to emphasize the complementary nature of WBE, traditional public health and clinical surveillance. Clinical observation of an infected patient for a rare condition or an increase in frequency of infections in the population for a common condition often proceeds meaningful signal in wastewater at a distant locality. For example, in the recent pandemic, SARS-CoV-2 in Wuhan, China and Lombardy, Italy were examples of what the rest of the world was going to experience. In this context, WBE can then be tailored to look for the appearance of the pathogen in other locations. In our network, for example, El Paso, TX is a border town that, because of its arid location, recycles nearly 100% of its water. It is also a global leader in wastewater technologies and a lead city for the monitoring program mentioned herein. Houston, on the other hand, is a major metropolis with many catchment areas and receives international travelers. In these cases, WBE can be used to catalyze development of a local response plan to an initially “far-off” threat. As the local response plan is initiated, WBE may activate alerts in nearby or more remote cities to initiate, intensify, or expand their monitoring programs. One might think of certain cities as canaries for a country (a good example for the U.S. might be large coastal cities that receive international travelers such as New York and Houston). Another way that WBE can be used following changes in reported cases is to answer the question of how broad the geographic distribution of an early outbreak is. For example, is the recent observation of a paralytic poliomyelitis case in NY a bellwether for reemergence of polio in the United States? Observations from clinical and public health surveillance does not necessarily have to precede WBE, and one reason to continue to improve WBE operations and laboratory sensitivity and specificity is to improve early detection before a local outbreak reaches the clinical horizon. The lead time examples of both polio and SARS-CoV-2 cases serve here to show that detection can significantly precede clinical detection.

Public health surveillance and action need to be seen as complementary and occurring simultaneously. For example, West Nile is a virus being monitored in our program. However, before West Nile is observed in an area, public health activities and educational programs should be working to eliminate standing water and implement other mosquito control measures. Second, what actions must be performed for the detection of less common but very concerning viruses? Following the 2014 occurrence of Ebola in Dallas, TX, protocol development, table-top exercises and personnel training for handling these and similar patients became a regular feature of public health, emergency management, first responders and health care. As we work to better understand how syndromic surveillance, healthcare case trend monitoring, WBE, and public health can better coordinate and communicate, it is important that WBE be included in the preparedness and response process with full knowledge of its strengths and weaknesses. Managing expectations is critical. At the moment, it is not certain if viruses of substantial concern such as Ebola or Smallpox can even be detected in public WW, and, if so, whether it will be a lead indicator of more transmissions.

**Figure 2:**
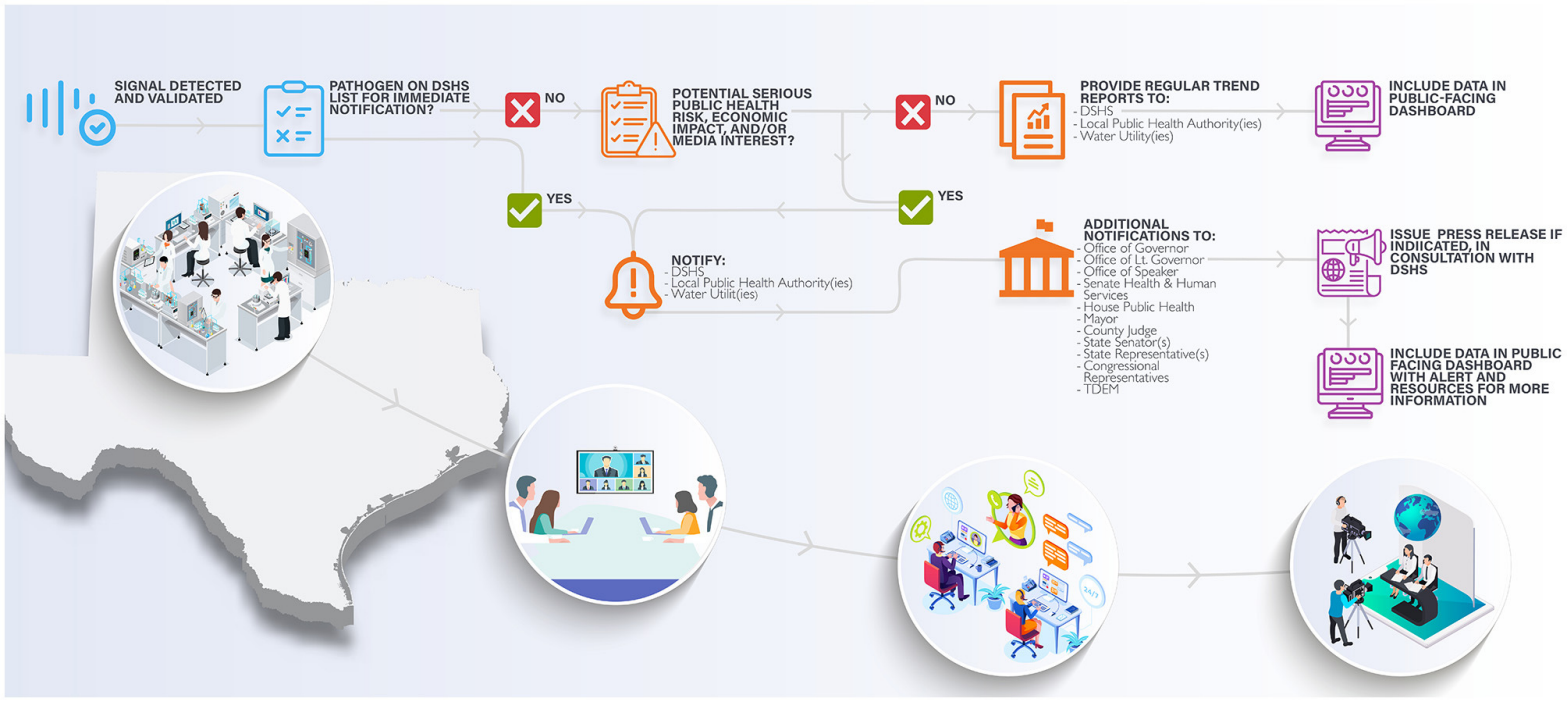
The wastewater detection notification scheme and network. Validated viral detection events of concern are assessed first for whether they are on the DSHS immediate notification list. DSHS as well as other stakeholders (local public health experts, state leaders, and/or utilities) are notified with additional action dependent on the threat of the concern (for example, smallpox vs. influenzae). Additional actions such as consultation with stakeholders and/or a press release may be needed. Viruses of seasonal or endemic nature (influenzae, SARS-CoV-2, RSV) are automatically entered into a real-time trend analysis with user-friendly reports provided on a regular basis (dependent on whether the targeted or agnostic method is being used). Long-term goals include a user-friendly community data set segmented in time, space, and viral species or variant detected.

The Consortium has identified four examples of public health and clinical actions informed by WBE that are of clear benefit, which include: (1) upstaffing in response to a detection event or trend. For example, during the COVID-19 pandemic it was not unusual for health care systems to employ contract nurses to meet surge capacity during a peak of the infection driven either by seasonality or emergence of novel SARS-CoV-2 variants. Similarly, wastewater-based forecasting has been used to aid in planning staffing needs and visitation policies at nursing home facilities; (2) In 2022, the United States had a summer monkeypox outbreak in multiple cities. At the time, vaccine supply was limited, so it was important to quickly develop strategies for getting the most population-benefit from the available orthopox vaccine. Mobile or pop-up vaccine units can be set-up in areas where the virus is present and at-risk individuals frequent; (3) related to 2 above, the Consortium is working to detect vaccine-preventable viruses such as measles and rubella. Given resistance against routine vaccination, it is possible viral WBE may report areas of vaccine fallout (for example, lowered vaccination rates leading to reemergence of vaccine-preventable diseases (of note, it is now reasonable to add SARS-CoV-2 to this list); (4) Finally, WBE can be a sturdy bridge to community engagement and education. An often over-looked but critical communication need is public awareness of WBE is presentation of a complex process into layperson digestible information, and what the information means for the community and its members (relative risk and behavior). For example, the Consortium aims to generate three types of data sets that scale in complexity depending on the stakeholders engaged. The first set will be highly technical, designed mostly for scientists developing the methodologies of detection and downstream analysis. The second will be a slightly less detailed but broad summary of levels aimed for public health and government officials. Finally, the last set includes community interactions (possibilities include dashboards, togglable links more in-depth information about threat levels or risk, etc) relevant to public health. This final set requires investigation into what is appropriate to report. Community engagement and buy-in (town halls) are critical, as some viruses can be stigmatizing to communities (for example, HIV). A summary of the Consortium's current plan for a public health arm following a validated detection event is shown in Figure 2.

## The future

Outside of the clear opportunities and challenges presented above with implementation of the TWC program, there are other exciting areas and challenges to consider as this field evolves and matures. On the detection front, the “what to detect” seems to be ever-changing and is always of importance. The U.S. Environmental Protection Agency has identified many pathogens that might be present in WW, including bacteria (*V. cholera, Salmonella typhi, enteropathogenic E. coli, Campylobacter jejuni, shigella dysinteriae*, and *Yersinia entrocolitica*), protozoa (Giardia, Cryptosporidium, *E. histolytica*), and helminths Ascaris, Ancylostoma, Trichuris, and Strongiloides (83). It is absolutely clear that WW harbors bacteria carrying genetic elements that confer resistance to antibiotics. This “silent pandemic” is expected to claim 10 million lives annually by 2050 and, since tracking of the emergence of resistant genes can prepare clinicians for where to provide antibiotic stewardship, WBE may be a useful source of such information. Although currently not considered by the Consortium, one also wonders if a community's immunologic status may be inferable from WBE, especially as it relates to inflammation or neutralizing antibody status to certain pathogens (one application of QMRA). On the public health front, it is clear relaying the information to stakeholders and the downstream steps they take are of substantial priority. This requires integration of utilities and their expertise in wastewater management, molecular scientists and technicians detecting the agents, the statistical and computer scientists analyzing the data, and the liaisons to connect these parties to the public health network, government officials and community. The Consortium is building a unified and integrated program that links these stakeholders in Texas. Finally, one wonders if the principles, pipeline, and program constructed from viral WBE may be applied to other medium monitoring activities, particularly air, which TEPHI plans to expand. Detection of viruses in air samples has already been demonstrated (84–87), but is associated with its own set of challenges, including sampling equipment (pump type, collection media), protocols (sampling volume, time, rate), varying sampling conditions (temperature, humidity), transport and storage conditions, and the type of virus to be detected (88).

In summary, the Consortium has begun efforts to implement a robust, real-time, and reliable viral WBE program across the state of Texas that brings utilities, microbiologists, chemists, clinicians, epidemiologists, statisticians, and public health experts together to identify and appropriately respond to viruses of pandemic potential. Some early success has been realized in utility onboarding, implementation of at least two molecular detection methods, and the creation of an integrated team that span the above fields of inquiry. In time, we hope to report further gains and obstacles in the coming year as the science and programmatic features of viral WBE continue to grow locally, nationally, and internationally.

## Data availability statement

The original contributions presented in the study are included in the article/Supplementary material, further inquiries can be directed to the corresponding authors.

## Author contributions

AM, JD, and JC conceived of the figures. AM, AT, JRC, VA, MT, BH, EB, KM, JR, CT, JD, and CB contributed to the writing and editing. The consortium designed the pipeline and framework for a statewide program. AM compiled all documents and organized the effort. JRC provided citation formatting. All authors contributed to the article and approved the submitted version.

## Supplementary material

Supplementary Figure 1 The molecular platform for viral wastewater detection and epidemiology. Wastewater samples are collected, shipped, and either immediately processed or stored. Large solids are sedimented and cleared supernatants are applied to electronegative filters for viral capture and nucleic acid extraction. Samples are then either tested by RT-PCR (“targeted” approach) or sent for library preparation and sequencing using a comprehensive human virus probe set (“agnostic” approach). The final stages include a statistical analysis of the data, examination of trends, and the production of a data report for health networks and the public.
