# Supplementary figures and images for: Wastewater pandemic preparedness: Toward an end-to-end pathogen monitoring program
Source: Front Public Health. 2023 Mar 21;11:1137881. doi: 10.3389/fpubh.2023.1137881 (PMC10070845; doi:10.3389/fpubh.2023.1137881)

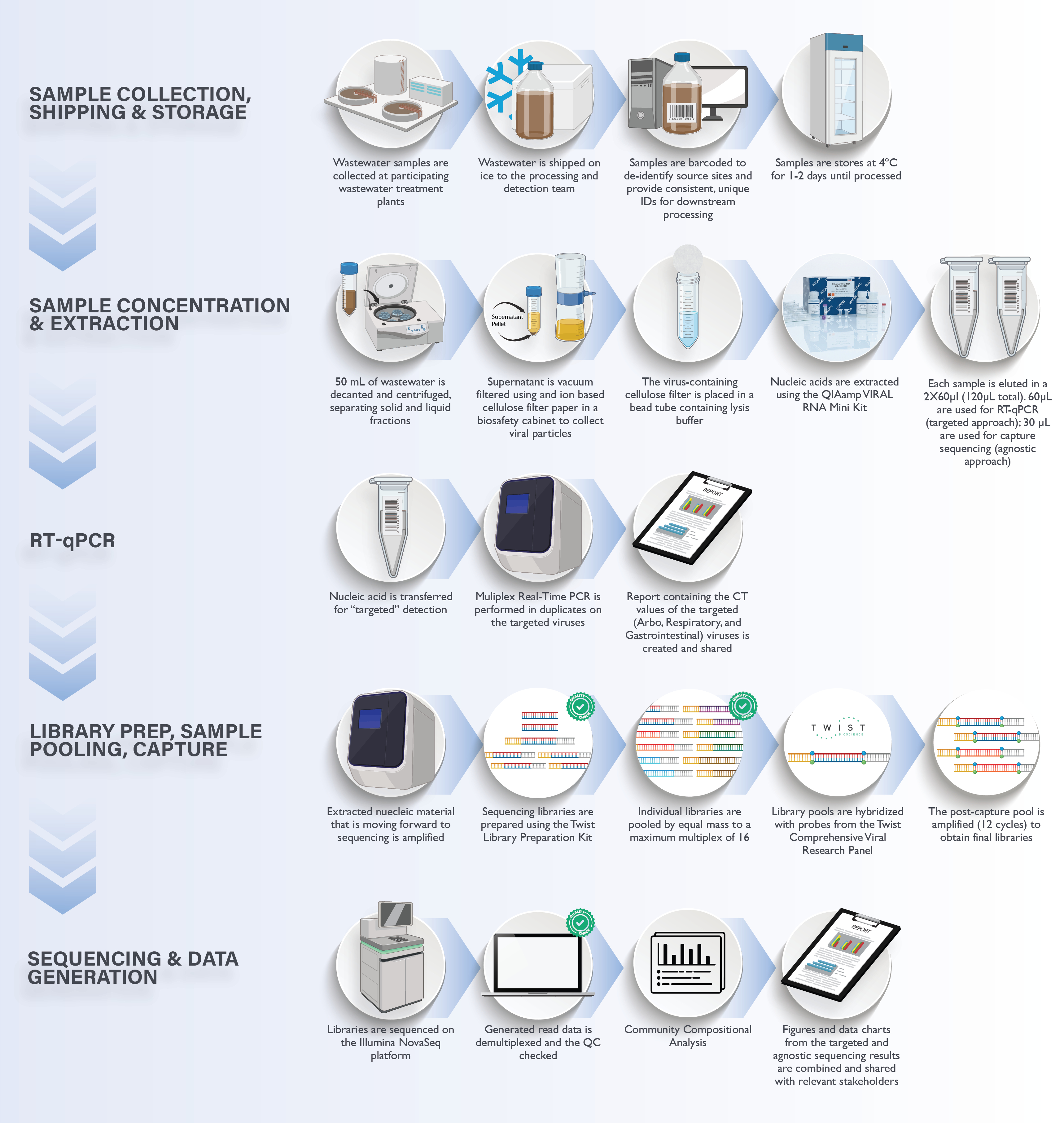

Supplement: Supplementary Figure 1 — The molecular platform for viral wastewater detection and epidemiology. Wastewater samples are collected, shipped, and either immediately processed or stored. Large solids are sedimented and cleared supernatants are applied to electronegative filters for viral capture and nucleic acid extraction. Samples are then either tested by RT-PCR (“targeted” approach) or sent for library preparation and sequencing using a comprehensive human virus probe set (“agnostic” approach). The final stages include a statistical analysis of the data, examination of trends, and the production of a data report for health networks and the public. [file Image_1.jpg]
