# Supplementary material for: Wastewater pandemic preparedness: Toward an end-to-end pathogen monitoring program
Source: Front Public Health. 2023 Mar 21;11:1137881. doi: 10.3389/fpubh.2023.1137881 (PMC10070845; doi:10.3389/fpubh.2023.1137881)
Supplement: Supplementary file 2 [file Table_1.docx]

**Supplemental Table 1: Advantages and disadvantages of the three approaches used by the consortium to monitor viral levels in wastewater**. The consortium’s terminology for each approach is indicated in italics with a specific description in parentheses. The advantages and disadvantages are framed relative to each of the three approaches.

| **APPROACH** | **ADVANTAGES** | **DISADVANTAGES** |
| --- | --- | --- |
| *Targeted Viral Detection*  (RT/Digital PCR) | ◦ High Sensitivity  ◦ Rapid turnaround (hours-days)  ◦ Low cost  ◦ High reproducibility  ◦ Increased automatability  ◦ Low technical know-how  ◦ Influenced less by reaction inhibitors (digital PCR) | ◦ Low Specificity (may miss viral variants/quasi species/strains)  ◦ Easier to contaminate |
| *Comprehensive Viral Detection*  (Probe-based sequencing) | ◦ High Specificity  ◦ Very comprehensive (hundreds to thousands of viruses – “the virome” captured)  ◦ Capture viral dynamics (“every signal relative to all other signals”)  ◦ May reveal novel, temporal, and global viral associations with human activity | ◦ Low Sensitivity (read and coverage dependent)  ◦ Less cost effective if sample number is low which may also lower turnaround time (weeks)  ◦ Reproducibility uncertain  ◦ Automatable, but more steps involved  ◦ High technical know-how  ◦ Computational analysis required  ◦ Detection dependent on probe library |
| *Targeted Viral Sequencing*  (whole-genome sequencing) | ◦ High Sensitivity  ◦ Very High Specificity (100% genome coverage possible)  ◦ Can be used to validate Targeted and Comprehensive approaches  ◦ Viral variants/quasi species/strains discernible  ◦ Single SNP resolution (track viral evolution) | ◦ Turnaround (days-weeks)  ◦ More costly  ◦ Highly specialized technical and technological know-how  ◦ Not quantitative to viral load |
