# Supplementary material for: Wastewater pandemic preparedness: Toward an end-to-end pathogen monitoring program
Source: Front Public Health. 2023 Mar 21;11:1137881. doi: 10.3389/fpubh.2023.1137881 (PMC10070845; doi:10.3389/fpubh.2023.1137881)
Supplement: Supplementary file 3 [file Table_2.docx]

**Supplemental Table 2**: **Some limitations and associated reasons that impact viral or pathogen detection**. Since each agent to be detected by WBE will have its own physical, chemical, and physiological properties, each agent will be impacted differently by wastewater conditions, sampling, processing, enrichment, and detection methodology.

| **Activity or Limitation to Consider** | **Reason to Consider** |
| --- | --- |
| Shed or excretion rate/properties | Virus or agent must be released in a form that is present in human excrement. Viruses that infect gastrointestinal or urogenital cells, or other cells that are eventually excreted, are thought to be primary sources of the virus in waste material. |
| Viral or nucleic acid stability | The structure and chemistry of the viral capsid may impact the viruses’ stability in complex and changing environmental conditions. Nucleic acid exposed to such conditions may also be subjected to degradation by RNase/DNases. |
| Capture | Regardless of the structure or chemistry of the viral capsid (or viral nucleic acid), the capture technique must be aligned with the physical and chemical properties of the virus surface. |
| Number of people infected/frequency of shedding | The concentration of the virus or agent will be in part determined by how many people are infected, how many people are served by the catchment area, and the frequency new virus is released by the infected host. |
| Transit time | The longer the transit time, the more time the viral agent can be negatively impacted by environmental or raw sewage conditions. |
| Wastewater composition | The chemical and physical composition of the wastewater may impact stability and detection of the viral agent. |
| Environment | Temperature, rainfall, pH, and so forth may all impact wastewater and, by extension, the stability and detectability of the viral agent in it. |
| Collection technique and sample storage | Volume of wastewater to be processed, the method of collection (grab versus time-based composite), shipping time (and environment shipped sample is exposed to), and storage conditions all may affect stability and detection of the viral agent. |
| Reaction inhibitors | Fragments of nucleic acid that may hybridize to and compete with primers or probes of the target of interest, or other small molecules that interfere with the PCR or sequencing reaction mechanisms. |
| Wastewater matter composition | Some viral agents would be expected to be associated with the supernatant fractions of cleared material; others may be bound to solids, be complexed to larger molecules, or be inside cells and therefore detected in the pellet or sedimented fraction. |
| Methodology conditions and optimization | Regardless of the method chosen for detection, its sensitivity and specificity will be determined by the primers or probes used in the assay, buffer conditions influencing those conditions, and the specific cycling or amplification times and temperatures, and so forth. Not all viral agents will share universal conditions optimal for detection. |
